# Tailoring Properties of Resol Resin-Derived Spherical Carbons for Adsorption of Phenol from Aqueous Solution

**DOI:** 10.3390/molecules26061736

**Published:** 2021-03-19

**Authors:** Karol Sidor, Tomasz Berniak, Piotr Łątka, Anna Rokicińska, Marek Michalik, Piotr Kuśtrowski

**Affiliations:** 1 Faculty of Chemistry, Jagiellonian University, Gronostajowa 2, 30-387 Kraków, Poland; karol.sidor@doctoral.uj.edu.pl (K.S.); tomasz.berniak@doctoral.uj.edu.pl (T.B.); piotr.latka@uj.edu.pl (P.Ł.); anna.rokicinska@uj.edu.pl (A.R.); 2 Institute of Geological Science, Faculty of Geography and Geology, Jagiellonian University, Gronostajowa 3a, 30-387 Kraków, Poland; marek.michalik@uj.edu.pl

**Keywords:** resol resins, carbonization, spherical grains, activated carbons, graphitization, heteroatom-containing carbon surface, adsorption of phenol, adsorption kinetics

## Abstract

The polycondensation of resorcinol and formaldehyde in a water–ethanol mixture using the adapted Stöber method was used to obtain resol resins. An optimization of synthesis conditions and the use of an appropriate stabilizer (e.g., poly(vinyl alcohol)) resulted in spherical grains. The resins were carbonized in the temperature range of 600–1050 °C and then chemically activated in an aqueous HNO_3_ solution, gaseous ammonia, or by an oxidation–reduction cycle (soaking in a HNO_3_ solution followed by treatment with NH_3_). The obtained carbons were characterized by XRD, the low-temperature adsorption of nitrogen, SEM, TGA, and XPS in order to determine degree of graphitization, porosity, shape and size of particles, and surface composition, respectively. Finally, the materials were tested in phenol adsorption. The pseudo-second order model perfectly described the adsorption kinetics. A clear correlation between the micropore volume and the adsorption capacity was found. The content of graphite domains also had a positive effect on the adsorption properties. On the other hand, the presence of heteroatoms, especially oxygen groups, resulted in the clogging of the pores and a decrease in the amount of adsorbed phenol.

## 1. Introduction

The growing demand for water is a consequence of the development of industry, increasing population and improving level of human life [1]. In developed countries, problems related to the quality of available water have been noticed for a long time. Despite efforts, water reservoirs in the European Union are still not free from serious pollutions. Their main sources are post-industrial and municipal wastes, as well as agricultural activities. On the European scale, only around 40% of surface water meets ecological requirements [2]. One of the main groups of harmful components found in surface waters are phenolic compounds, which exhibit toxic properties that cause serious short- and long-term adverse effects for humans and animals [3,4]. These compounds tend to accumulate in the environment, which additionally increases their harmfulness. The sources of phenolic pollution can be divided into natural and anthropogenic. Natural sources include processes of organic matter decomposition, as they are produced by some microorganisms and plants [5]. On the other hand, human activities contribute to the emission of phenols, mainly due to the chemical industry (which produces and processes phenolic resins), wood manufacturing, the construction industry, and the production of dyes, textiles, explosives, and plastics [6]. Phenolic pollutants released from agriculture are mainly pesticides, herbicides, and insecticides [7]. Phenols are also found in municipal wastewater as a result of use of products such as soap, creams, deodorants, and varnishes [8,9].

Many efficient methods for the elimination of phenolic compounds have been developed, which may be helpful in achieving the appropriate purity of water introduced to surface water reservoirs. These include photocatalytic degradation, ozonation, extraction, biodegradation, adsorption, membrane separation, and ion exchange [10]. However, the most important techniques commercially used in the wastewater treatment are ozonation and adsorption on activated carbons [11]. The involvement of carbon adsorbents brings additional benefits due to the possibility of recovering phenols and reusing them in various applications.

It is commonly believed that the adsorption of phenolic compounds on carbon materials can proceed through (1) the dispersion interaction of π electrons of a phenolic ring with π electrons of a graphene layer [12], (2) electron donor–acceptor complex formation between donor groups on a surface (e.g., carbonyl groups) and a phenol molecule acting as an acceptor [13], and (3) hydrogen-bond formation between a hydroxyl group of phenol and oxygen-containing surface groups [14,15]. The efficiency of the phenol adsorption process is influenced by many parameters, such as the concentration of the pollutant, pH of the solution, temperature, and presence of other ions [16]. However, adsorption capacity depends also on many different physicochemical properties of a carbon adsorbent, including the degree of graphitization, the presence of heteroatoms, and the density of functional groups on its surface. The presence of nitrogen functionalities, increasing surface alkalinity, enhances the affinity of weakly acidic phenol molecules to the surface of activated carbon [15]. Furthermore, phenol adsorption is influenced by the pore size distribution of a carbon adsorbent [17]. Hsieh et al. [18] showed that a shift of average micropore size towards wider diameters results in a decrease in the adsorption capacity per unit area while increasing the adsorption strength [19].

Carbon adsorbents are manufactured from a wide variety of high-carbon raw materials, including coal, lignite, wood, peat, coconut shell, lignin, petroleum coke, and synthetic polymers. A carbon material precursor should have a high density, hardness, contain a small amount of inorganic matter, and be widely available and cheap [20,21]. Producing carbon adsorbents is based on two main stages: (1) the carbonization of a raw material at temperatures above 550 °C at an inert atmosphere and (2) the physical or chemical activation of the obtained carbon [22]. Various types of resol resins (e.g., phenol–formaldehyde, resorcinol–formaldehyde, melamine–formaldehyde, and urea–formaldehyde resins) are particularly desirable for the production of highly microporous activated carbons, which can be used as adsorbents for a gas and liquid phase treatment [23,24]. Spherical resol resins based on resorcinol and formaldehyde can be synthesized using sol–gel polycondensation in the modified Stöber method [25].

Most of the synthesized commercial carbon adsorbents are available in the form of powder or granules. However, it should be emphasized that the morphology of the carbon adsorbent plays a key role in the kinetics of the adsorption process. The pulverization of the carbon granulates often allows one to significantly improve the adsorption rate, especially when dealing with large pollutant molecules [26]. Despite the high specific surface area and content of functional groups on the surface, powder adsorbents are not suitable materials for use in continuous operations. Columns packed with such materials can be ineffective due to pressure drops related to the internal resistance of an adsorbent bed. Consequently, the efficiency of such a system decreases, which increases operating costs [27]. A potential solution to this problem is the implementation of a spherical grain-shaped bed, as in the case of high-performance liquid chromatography [28]. The application of spherical activated carbons improves mechanical and physicochemical properties compared to classic materials with a non-regular particle shape [29]. By an appropriate selection of synthesis conditions, it is possible to produce spherical activated carbons with a large volume of micropores and a controlled distribution of their size [30]. Grains with a size in the sub-micro region tend to coagulate, resulting in larger carbon aggregates [24,31]. The aggregation of the particles causes the formation of mesopores in the adsorbent structure. Their presence allows for a stream of pollutants to more effectively penetrate the carbon particles. As a consequence, the faster saturation of the bed can be achieved in such an adsorption system [32].

Considering the promising adsorption properties of spherical carbons derived from resol resins, we decided to develop a facile method of their production. The aim of the presented work was to analyze the influence of a wide range of synthesis conditions on the size, shape, porosity, degree of graphitization, and distribution of surface functional groups containing heteroatoms (oxygen and nitrogen) of the formed grains. Planned in this way, the research allowed us to determine the effect of the above-mentioned parameters on the adsorption capacity in the removal of phenol from an aqueous solution. The collected results shed new light on the mechanism of the phenolic compounds’ adsorption process and the key features of resol resin-derived carbon adsorbents that determine their adsorption performance.

## 2. Results and Discussion

### 2.1. Effect of Grain Size and Morphology

**Figure 1:**
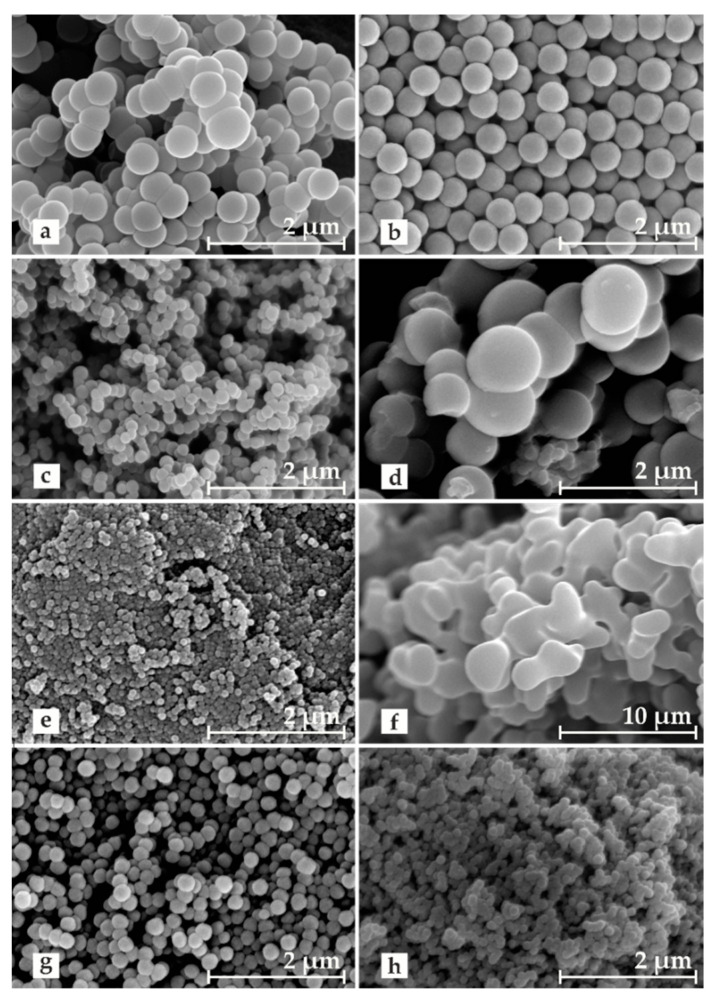
SEM pictures for carbonized resol resins: (**a**) 52_non, (**b**) 52_PVA, (**c**) 52_PVP, (**d**) 52_X100, (**e**) 52_P123, (**f**) 52_HCl, (**g**) 61_PVA, and (**h**) 131_PVA.

As the results of our research showed, by selecting appropriate Stöber synthesis parameters, such as the type of stabilizer, the type of initiator, and the solvent composition (i.e., the volume ratio of water to ethanol) in the reaction mixture, the morphology of the formed resol resin grains can be controlled in a wide range. The relative sphericity of the grains was strongly influenced by the use of proper stabilizer during the polycondensation of resorcinol and formaldehyde. This effect was revealed by the SEM images collected for the carbon materials after the carbonization step (Figure 1a–e).

Since the polycondensation process took place in the stabilized emulsion droplets, their size and dispersion (controlled by the presence of the stabilizer) played key roles in the formation of spherical grains and their potential aggregation. In the case of the carbon materials—52_non (Figure 1a) and 52_X100 (Figure 1d)—where the average grain size was above 600 nm, strong coagulation was observed, and individual grains joined into characteristic bead-like structures. For the materials synthesized in significantly smaller droplets, no clear irregularities in the shape and size of grains were found, but they were much more prone to aggregation, which was especially visible for the 52_P123 material (Figure 1e). Compared to all the obtained carbon materials, the sample stabilized with PVA was quite different (Figure 1b). The 52_PVA grains were characterized by a low coefficient of polydispersion. Their individual shape was practically perfectly spherical, and they were not observed to stick together into larger structures. The surprisingly favorable stabilizing effect obtained with PVA can be explained by a dehydration polycondensation reaction between a large amount of hydroxyl groups in the stabilizer and hydroxymethyl groups in the resol resin [33].

Following this path, we synthesized stable PVA resorcinol–formaldehyde resins under various pH conditions (alkaline or acidic) to change the composition of the water–ethanol solvent. After analyzing the SEM images (Figure 1b,f–h), it was confirmed that the use of ammonia as the polycondensation initiator played a beneficial role in the formation of spherical grains [25]. The replacement of ammonia by HCl destabilized the system due to the lack of hydrogen bonds between the solvent molecules and the monomer molecules, which resulted in the obtained resol resin grains strongly aggregating to form quickly-precipitating micrometric conglomerates of oblong shape. The decrease in the ethanol content in the NH_3_-initiated reaction system resulted in a reduction of the droplet size formed in the reaction emulsion. As shown in Table 1, the materials prepared under such conditions, i.e., 61_PVA and 131_PVA, exhibited a much smaller grain size (approximately 230 and 100 nm, respectively) compared to the previously discussed 52_PVA (approximately 460 nm). Reducing the size of grains resulted in a deterioration of their sphericity. This effect was especially noticeable for the 131_PVA sample, for which additionally strong grain aggregation was observed.

**Figure 2:**
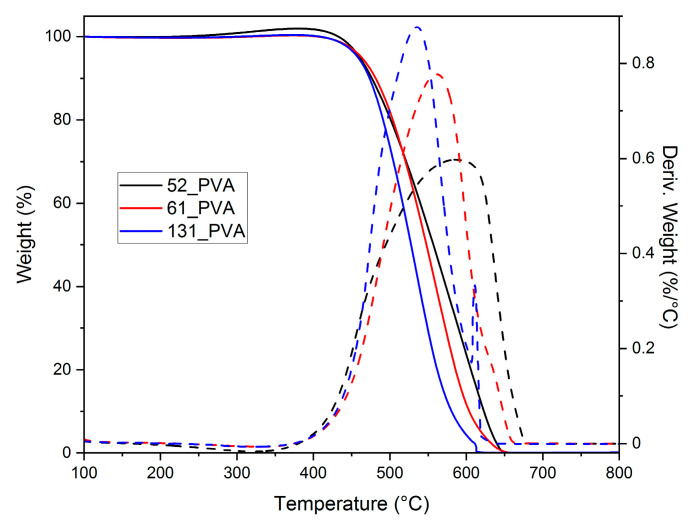
TG (solid) and DTG (dashed) curves recorded in flowing air for the carbon materials of various grain sizes synthesized by the carbonization of PVA-stabilized resole resins at 600 °C.

Thermogravimetric analysis in oxidative atmosphere showed that the size of carbon material grains significantly affected their thermal stability (Figure 2). After raising their average diameter from 100 to 460 nm, a shift in temperature of the maximum burning rate from 535 to 590 °C was found. This could be explained by the cascade ignition mechanism. Smaller grains burned at lower temperatures, initiating the ignition of other grains.

**Figure 3:**
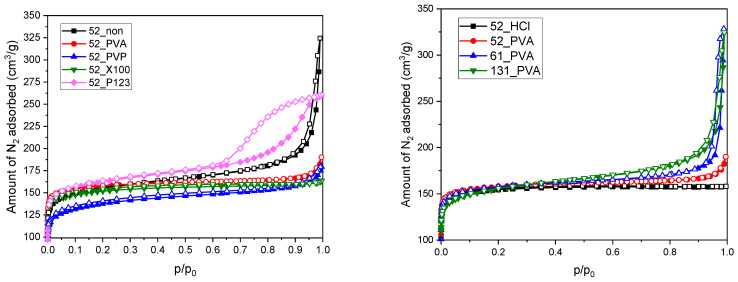
N_2_ adsorption–desorption isotherms measured for the studied carbon materials carbonized at 600 °C.

The results of low-temperature nitrogen adsorption for the prepared resol resin-derived carbon materials are presented in the form of the appropriate isotherms in Figure 3 and the determined textural parameters in Table 1. The shape of the isotherms could be described as modified type I according to the International Union of Pure and Applied Chemistry (IUPAC) classification, which reveals the significant participation of micropores in the porous structure of the synthesized carbonizates. One difference in the standard shape of the type I isotherm was the increase in the amount of adsorbed nitrogen at the highest relative pressures (p/p_0_ > 0.8) corresponding to the formation of macropore system in the material structure. This could be attributed to voids between aggregated grains, which were particularly evident for the sample obtained in the absence of stabilizer (52_non), as well as for the materials based on PVA-stabilized resol resins that were synthesized in the presence of a high excess of water in relation to ethanol (61_PVA and 131_PVA). Moreover, it should be noted that the porous properties of the 52_P123 material significantly differed from the others. The nitrogen adsorption–desorption isotherm for this sample shows a hysteresis loop above relative pressures p/p_0_ ~ 0.6, which allows us to classify it as type IV. Such an isotherm shape is characteristic of mesoporous materials. The isotherm collected for 52_P123 in combination with the corresponding SEM images (Figure 1e) confirmed the presence of slit pores between strongly structured clusters of spherical grains.

Regardless of the synthesis conditions, the specific surface areas of the spherical resol resin-derived carbons determined by the Brunauer–Emmett–Teller (BET) method were in the range of 510–640 m^2^·g^−1^ (surface areas calculated using the Langmuir model—600–740 m^2^·g^−1^) (Table 1). On the other hand, significantly greater differences were observed for the total pore volumes, which were the highest for 61_PVA and 131_PVA (approximately 0.5 cm^3^·g^−1^) due to the previously described effect of grain aggregation. Such an origin of this effect was confirmed by the similar micropore volumes for all tested materials (values within the range of 0.18–0.23 cm^3^·g^−1^), which clearly confirmed the presence of larger interparticle voids. The determined textural parameters were similar to the results reported by other researchers for resol-based carbon materials, which typically have BET surface areas within the range of 450–750 m^2^·g^−1^, regardless of morphology [34,35,36,37].

**Figure 4:**
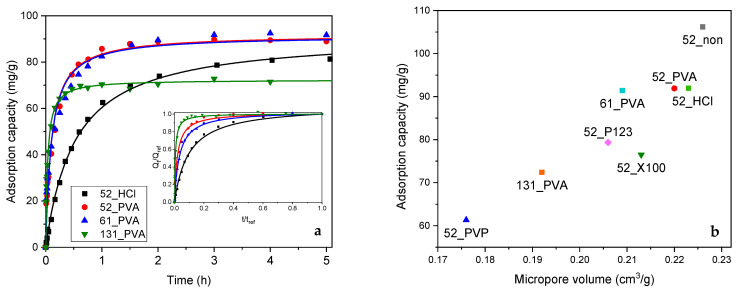
(**a**) Results of adsorption tests in phenol removal from an aqueous solution with fitting by both standard and dimensionless pseudo-second order (PSO) model. (**b**) Relationship between the adsorption capacity and volume of micropores for the studied carbon materials carbonized at 600 °C.

Adsorption tests confirmed that the carbon materials obtained by carbonization at 600 °C were efficient adsorbents of phenol from a water phase. Two models were used to describe the kinetics of the studied adsorption process—the pseudo-first order model and the pseudo-second order model. The determined parameters for both chosen models are presented in Table 2. On the basis of the R^2^ coefficient, it should be indicated that the pseudo-second model better described the phenol adsorption on the resol resin-derived carbon materials. The grain sphericity strongly influenced the pseudo-second order adsorption rate constant ($k_{ad}^{\prime \prime }$). The 52_X100 material, characterized by an irregular sphericity, strong aggregation, and high polydispersity, showed the lowest value of $k_{ad}^{\prime \prime }$ among the investigated materials. The 52_non material, which showed an improved sphericity and improved monodispersion with strong single grain aggregation, had a higher adsorption rate constant compared to 52_X100. For highly spherical and monodisperse materials, i.e., 52_PVA and 52_PVP, high $k_{ad}^{\prime \prime }$ values of approximately 3 times greater than for the 52_non carbon material were observed. The highest value of $k_{ad}^{\prime \prime }$ was obtained for 52_P123. This was related to the presence of mesopores in the structure of this material, which facilitated the migration of phenol molecules inside the aggregated material. The size of the formed grains also influenced the adsorption rate—the smaller the grains, the faster the process (Table 2 and Figure 4a). An exceptionally high value of $k_{ad}^{\prime \prime }$ was observed for the 131_PVA sample, which must be combined with two parameters of this material, namely grain size and pore volume. A deeper analysis showed that the values of dimensionless pseudo-second order (PSO) coefficient $\left(R_{w}\right)$ correlated well with the mean grain sizes for 131_PVA, 61_PVA, 52_PVA, 52_non, and 52_HCl (the obtained linear regression gave a fitting coefficient R^2^ = 0.9744). This result confirmed that the grain size had a significant influence on the kinetics of phenol adsorption on the resol resin-derived carbon materials.

From the application point of view, apart from the adsorption kinetics, adsorption capacity is very important. It should be noted that the 52_non material, despite its low value of $k_{ad}^{\prime \prime }$, was characterized by the highest efficiency in the removal of phenol. This can be explained by easy accessibility of the grain surface. The proper selection of the stabilizer has a significant impact on the operation of the corresponding carbon material. Poly(vinyl alcohol) appeared to be the most optimal stabilizer.

A very interesting correlation between the determined adsorption capacity of the tested materials and the micropore volume was found (Figure 4b). Along with an increase in the value of this textural parameter, an increase in adsorption capacity was observed. This may indicate the key role of the micropore system on the effectiveness of phenol elimination from an aqueous solution. Similar relationships were previously described for other activated carbons [38,39,40]. Generally, the presence of microporosity enhances interaction between the π electrons in the phenolic ring of phenol and the basal plane of carbon due to the greater potential energy [41]. As a result, phenol adsorption, which was well-fitted by the pseudo-second order kinetics model, occurs more efficiently. Nevertheless, the spherical shape of grains, as shown above, plays a beneficial role in the adsorption process because it significantly facilitates the access of adsorbate molecules to the interior of the micropores.

### 2.2. Effect of Graphitization

**Figure 5:**
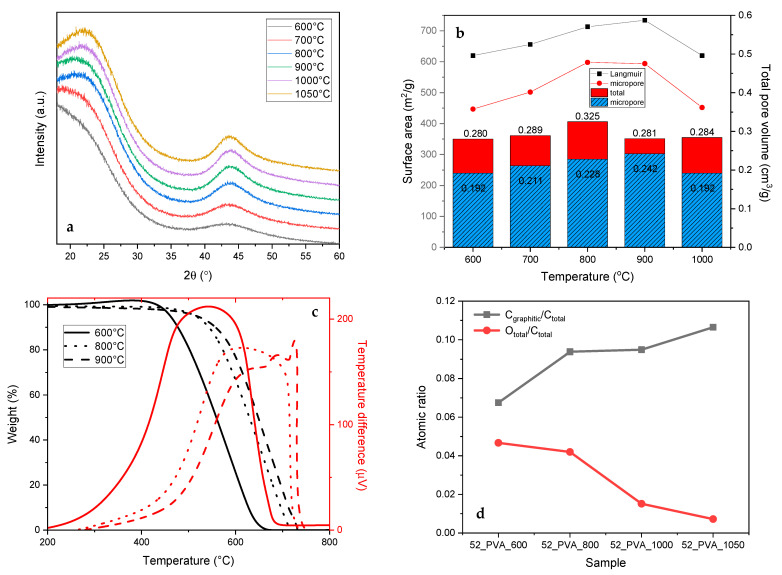
Changes in properties of the 52_PVA resin-derived carbon materials induced by raising the carbonization temperature: (**a**) X-ray diffraction patterns, (**b**) textural parameters, (**c**) TG–DTA curves, and (**d**) surface composition determined by XPS.

In a further study, we focused on the 52_PVA resol resin as the carbon precursor with the expected morphology, which was carbonized at different temperatures. The yields of carbonization were 60% (600 °C), 50% (700 °C), 48% (800 °C), 47% (900 °C), and 46% (1000 °C). It was found that increasing carbonization temperature resulted in changes related to the structure of the produced activated carbons. The most important parameter that varies during annealing at higher temperatures is the degree of graphitization. The presence of graphite-like domains in the final samples was manifested in the XRD patterns by the diffraction peaks at approximately 22° and 44° (Figure 5a), the intensities of which increased with the carbonization temperature. Arranging the graphite-like structure initially improved the textural parameters—the specific surface and the volume of micropores (Figure 5b). Only after exceeding 1000 °C the specific surface area and the volume of micropores begin to be reduced. The greater participation of the crystalline graphite-like structure made the carbon material much more thermally stable. As can be seen in the TG and DTA curves (Figure 5c), the burning temperatures of the carbon materials gradually shifted to higher values with the increasing carbonization temperature.

The re-arrangement of the amorphous carbon to the crystalline graphite-like form was particularly noticeable for the sample that was thermally treated at 1050 °C, for which the proportion of graphite carbon in the total amount of carbon (C_graphitic_/C_total_) on the surface, determined by XPS, exceeded 10% (Figure 5d). The higher temperature of the thermal treatment simultaneously caused the removal of heteroatoms (N and O) from the carbon surface. This was evidenced by the O_total_/C_total_ surface ratio shown in Figure 5d, which decreased from 0.05 (carbonization at 600 °C) to 0.01 (carbonization at 1050 °C).

**Figure 6:**
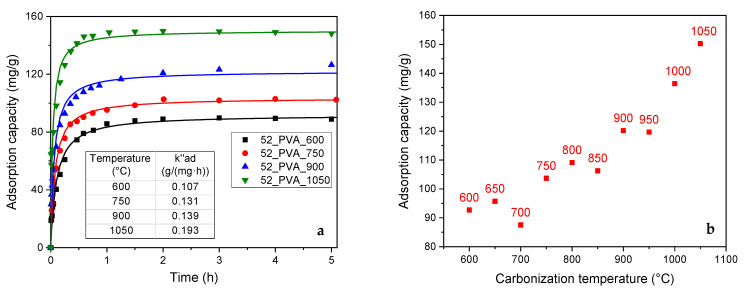
(**a**) Results of adsorption tests in phenol removal from an aqueous solution with fitting by the PSO model. (**b**) Relationship between adsorption capacity and carbonization temperature for the 52_PVA resin annealed at different temperatures.

The increase in the content of the graphite-like crystalline phase in the resol resin-derived carbon materials also significantly improved their adsorption properties (Figure 6a). On the one hand, the adsorption capacity ($Q_{max}$) rose from about 90 mg·g^−1^ (the 52_PVA samples carbonized at 600–700 °C) to 150 mg·g^−1^ (52_PVA_1050). On the other hand, saturation during the adsorption process was much faster. The time needed to reach the equilibrium adsorption capacity in the case of the material with the highest degree of graphitization was almost twice shorter compared to that for the most amorphous carbon material (52_PVA_600).

The clear correlation between the adsorption capacity and the degree of graphitization confirmed the previous discussion on the phenol adsorption mechanism. It should be noted that raising the carbonization temperature favored the formation of micropores, in which the process preferentially took place. Additionally, larger amounts of graphite domains were created under these conditions. The interaction of the graphite surface with the π electrons of phenol molecules was crucial in enhancing the adsorption effect.

The adsorption capacity of the 52_PVA carbon was additionally compared to commercial activated carbon WG-12 (S_BET_ = 1064 m^2^·g^−1^; V_micro_ = 0.375 cm^3^·g^−1^) used for water remediation. The adsorption parameters determined for this sample according to the PSO model were as follow: $Q_{max}$ = 119 mg·g^−1^, $k_{ad}^{\prime \prime }$ = 0.01 g·mg^−1^·h^−1^, and R^2^ = 0.8965. Thus, our carbon materials (pyrolyzed at higher temperatures) exhibit significantly better performance.

### 2.3. Effect of Surface Modification

**Figure 7:**
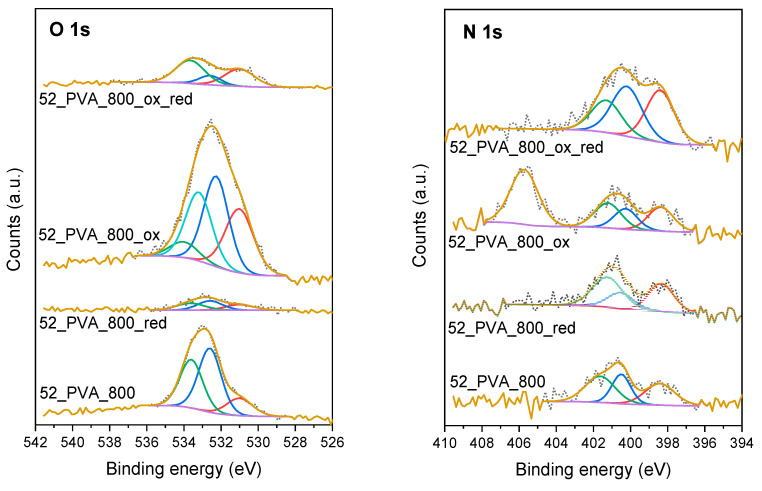
High-resolution O 1s and N 1s XPS spectra for the resol resin-derived carbon materials carbonized at 800 °C and subsequently modified in an oxidizing/reducing atmosphere.

Other parameters analyzed as potentially affecting the adsorption properties were the type and concentration of heteroatom-containing functionalities distributed on the surface of 52_PVA. Considering the significant influence of the carbonization temperature on the degree of graphitization, in this case, three materials carbonized at different temperatures (600, 800, and 1050 °C) were selected. It was obvious that the carbonized resol resin samples had a certain amount of surface heteroatoms, but these materials were additionally modified by treatment with nitric acid solution (“ox” series), gaseous ammonia (“red” series), or oxidation with HNO_3_ and subsequent reduction with NH_3_ (“ox_red” series). After such treatments, we investigated the surface composition using XPS. Figure 7 demonstrates examples of XPS O 1s and N 1s spectra for the 52_PVA sample carbonized at 800 °C before and after the modifications.

It is clearly visible that the chosen modification method had a huge impact on the type and concentration of oxygen- and nitrogen-containing groups determined on the surface of the materials. Basically, three forms of surface oxygen were identified in the XPS O 1s spectra. The peaks at binding energies of 531.0 ± 0.2, 532.4 ± 0.2, and 533.8 ± 0.2 eV corresponded to C=O, O=C-O, and C-O, respectively [42,43,44]. In turn, in the XPS N 1s spectra, three peaks at 398.6 ± 0.3, 400.2 ± 0.2, and 401.5 ± 0.2 eV were distinguished as a result of deconvolution. They could be attributed to the presence of pyridinic N, pyrrolic N, and graphitic N, respectively [45,46,47]. Interestingly, in the case of the samples exposed to the nitric acid solution (“ox” series), additional components exceptionally appeared in the XPS spectra, i.e., at 533.2 ± 0.2 eV for O 1s and 404.4 ± 0.3 eV for N 1s. They obviously indicated the formation of nitro groups on the surface, which disappeared after the following reduction with ammonia [48].

The exact contents of various types of heteroatoms on the surface of the studied samples are presented in Table 3. Generally, the demonstrated data confirmed that the amounts of oxygen- and nitrogen-containing groups decreased with increasing carbonization temperature. The oxygen functionalities could, however, be successfully restored by treatment with the nitric acid solution. It should be noted that the carbonization temperature played a significant role in this regard. Most surface oxygen-containing groups appeared after the contact of the 52_PVA_600 material with HNO_3_, while the least appeared for 52_PVA_1050. This was not surprising due to the progressive degree of graphitization, which hindered the formation of surface functionalities containing heteroatoms. This effect mainly applied to carboxyl (O=C-O), phenolic (C-O), and nitro (N-O) groups, because for quinone moieties (C=O), regardless of the carbonization temperature, a comparable quantities (2.3–2.4 at%) formed after the treatment with HNO_3_. The action of the reducing agent (namely gaseous NH_3_), both for freshly carbonized samples and those subjected to subsequent modification in the HNO_3_ solution, resulted in a noticeable loss of oxygen-containing groups. Only a little more was left in the case of the previously oxidized materials, especially those annealed at lower temperatures (e.g., 52_PVA_600).

The 52_PVA resol resin-derived materials carbonized at 600 and 800 °C contained 1.4–1.5 at.% of N on their surface. Only raising the carbonization temperature to 1050 °C caused a disappearance of the nitrogen-containing groups to the level of 0.6 at.% of N. The treatment of the carbons in gaseous ammonia did not actually change the N distribution, except for graphitic species, the content of which slightly increased, especially in the case of 52_PVA_1050. On the other hand, activation in the HNO_3_ solution affected the generation of NO_2_ groups. Interestingly, the nitrogen of these groups remained in the materials after subsequent reduction with NH_3_, creating a wide spectrum of different N species (both pyridine, pyrrole, and graphitic). The 52_PVA_600 material was the most saturated with these groups. Forming pyridinic and pyrrolic functionalities by the reduction of NO_2_ groups dispersed on mesoporous carbon obtained by the nanoreplication of silica gel was previously reported by Szewczyk et al. [49].

Table 4 summarizes the textural parameters of the studied materials. It can be seen that the activation of the samples carbonized at 600 and 800 °C resulted in a reduction of their porosity and, consequently, their specific surface area. This effect was particularly noticeable after contact with the HNO_3_ solution, as a result of which a large number of oxygen-containing surface groups were formed. Their presence most likely blocked relatively narrow micropores. After removing the oxygen functionalities by the reducing agent, the pore system was re-opened and the original textural parameters are restored. In this context, the sample carbonized at 1050 °C with a high degree of graphitization behaved differently. The alignment towards the graphite domains led to the accumulation of amorphous carbon material in the pores after carbonization, which was effectively removed by contact with the HNO_3_ solution and gaseous ammonia. Hence, the activation of 52_PVA_1050 had very positive effects by increasing the volume of micropores and the specific surface area.

The various surface-modified carbon materials derived from the 52_PVA resol resin were finally tested as adsorbents in the phenol removal. The obtained kinetic results were fitted with the pseudo-second order model, and the relevant parameters calculated on the basis of this model are summarized in Table 5.

It should be noted that the treatment of the carbon material with nitric acid, regardless of the carbonization temperature, resulted in a very significant degradation of its adsorption properties, which was not only attributed to reduction in the volume of micropores but also to the presence of groups unfavorable in terms of surface interactions. Unlike the materials activated in the HNO_3_ solution, the reduction of spherical 52_PVA carbon materials in the NH_3_ stream caused smaller changes in the adsorption capacity. The deterioration of the adsorption capacity was especially noticeable for the material for which pyrolysis was carried out at the highest temperature. On the other hand, the adsorption tests in the elimination of phenol from the water phase on the materials after the ox_red cycle showed unequivocally improved adsorption properties compared to the carbon materials modified in the one-stage process. The application of the ox_red cycle resulted in the reduction of the adsorption capacity by about 20% in relation to the starting material. It should be assumed that the incorporation of nitro groups into the surface structure of the carbon material restricted the access of adsorbate molecules to the interior of micropores. The transformation of the NO_2_ groups into the various N forms built into the carbon matrix caused the micropores with graphite planes localized inside to become re-opened. Internal adsorption centers were thus accessible to phenol molecules, but the presence of nitrogen moieties did not favor adsorption.

Therefore, when analyzing the above results, it should be concluded that the presence of surface heteroatoms (O and N) did not have a beneficial effect on the adsorption process, which ran preferentially on graphite surfaces, mainly in micropores.

## 3. Materials and Methods

### 3.1. Chemicals

All used chemicals and their origins are as follows: resorcinol (Chempur, Piekary Śląskie, Poland, pure p.a.), formaldehyde (36–38%, POCH, Gliwice, Poland, pure p.a.), ethanol (98%, Chempur, Piekary Śląskie, Poland, pure p.a.), poly(vinyl alcohol) (MW = 72000, POCH, Gliwice, Poland), polyvinylpyrrolidone (PVP40, Sigma-Aldrich, China), poly(ethylene glycol)-block-poly(propylene glycol)-block-poly(ethylene glycol) (Pluronic P123, Sigma-Aldrich, USA), polyethylene glycol tert-octylphenyl ether (Triton X100, Sigma-Aldrich, USA), ammonia solution (28–30%, J. T. Baker, USA), hydrochloric acid solution (37%, Honeywell, USA, puriss p.a.), nitric acid solution (65%, Sigma-Aldrich, China, puriss p.a.), phenol (Chempur, Piekary Śląskie, Poland, pure p.a.), gaseous ammonia (20% NH_3._, 80% He, Air Products, Belgium), nitrogen (5.2, Air Products, Świerzewo, Poland), and commercial activated WG-12 carbon (Gryfskand, Hajnówka, Poland).

### 3.2. Synthesis and Modification

Resol resins were prepared b they polycondensation of resorcinol and formaldehyde in a water–ethanol mixture using the modified Stöber method [25]. Briefly, a typical synthesis was carried out in a 1 L three-necked flask equipped with a thermometer, a spiral condenser, and a mechanical stirrer. The flask was placed in an oil bath on a heating plate. Before starting polycondensation, formaldehyde (7 mL), resorcinol (5.0 g), a water–ethanol mixture (700 mL), and a suitable stabilizer were introduced into the flask. The resulting mixture was mixed (200 rpm) at 40 °C. After 30 min, an ammonia solution (or, alternatively, a HCl solution) was added to initiate polycondensation, and the reaction mixture was left at 40 °C for 24 h. Subsequently, the temperature was increased, and the obtained gel was aged at 100 °C for another 24 h. The detailed compositions of the reaction mixtures used during the syntheses of resol resins are shown in Table 6.

The prepared sample was centrifuged using the MPW-352 centrifuge (MPW, Warsaw, Poland) (5000 rpm) for 45 min, and the obtained solid was dried at 60 °C for 16 h. The material was then finely ground using an agate mortar and subjected to further drying at 60 °C for 24 h.

Finally, the synthesized resol resins were carbonized in a tube furnace under a nitrogen flow (60 mL·min^−1^) to obtain the corresponding carbon materials. The resins were thermally treated at temperatures within the range of 600–1050 °C for 4 h (with a temperature ramp of 1 °C·min^−1^). The temperature used during the carbonization of a given carbon material was coded in a sample name. For example, the 52_PVA resin carbonized at 600 °C is denoted as 52_PVA_600.

Selected resin-derived carbons were modified in three different ways:

1. Oxidation with a concentrated HNO_3_ solution was done according to the following procedure: 1 g of a carbon was treated in 10 mL of the oxidant solution at 50 °C for 3 h, e.g., the HNO_3_ treated 52_PVA_600 sample is denoted as 52_PVA_600_ox.
2. Reduction with gaseous ammonia was done with (i) heating to 250 °C in flowing N_2_ (heating rate of 20 °C·min^−1^ and total flow rate of 100 mL·min^−1^), (ii) the desorption of surface impurities at 250 °C for 30 min (total flow rate of 100 mL·min^−1^ N_2_), (iii) heating to 500 °C (heating rate of 20 °C·min^−1^ and total flow rate of 100 mL·min^−1^), and (iv) reduction under the ammonia atmosphere at 500 °C for 1 h (total flow rate of 30 mL·min^−1^), e.g., the NH_3_ treated 52_PVA_600 sample is denoted as 52_PVA_600_red.
3. Oxidation with concentrated HNO_3_ solution followed by reduction with gaseous ammonia was done using the same conditions as in modifications 1 and 2, e.g., the oxidized and further NH_3_ treated 52_PVA_600 sample is denoted as 52_PVA_600_ox_red.

In the reduction steps (modification 2 and 3), a quartz flow microreactor was used. An amount of 450 mg of a sample was introduced onto a quartz wool plug located in the middle of the reactor, and the bed temperature was controlled by a thermocouple coupled with a temperature controller and an electric oven.

### 3.3. Characterization

The structure of the carbon samples was determined by XRD. The XRD patterns were collected on a Bruker D2 Phaser instrument (Bruker, Billerica, MA, USA) using Cu Kα radiation (λ = 1.54184 Å) and a LYNXEYE within a 2θ range of 5–60° at a step of 0.02°.

The textural properties of the materials were studied by low-temperature adsorption of nitrogen at −196 °C using a Micromeritics ASAP 2020 sorptometer (Micrometrics, Norcross, GA, USA). The adsorption measurement was preceded by an outgassing procedure at 250 °C for 6 h under vacuum. Specific surface areas were calculated using the BET and Langmuir models. Single point adsorption at p/p_0_ → 1 was used to obtain total pore volumes. Micropore and mesopore volumes were determined using the t-plot model (multipoint fitting in p/p_0_ = 0.1–0.3) and the Barrett-Joyner-Halenda (BJH) model (assuming a pore diameter range of 2–50 nm), respectively.

Morphology was investigated by SEM imaging using a Hitachi S-4700 field emission scanning electron microscope (Hitachi, Toyko, Japan) at an accelerating voltage of 20 kV. Samples were mounted on sticky carbon discs and coated with a gold layer. The secondary electrons (SE) signal was used for observations.

Thermal analyses were done using an SDT Q600 thermobalance (TA Instruments, New Castle, DE, USA). An amount of about 10 mg of a sample was introduced into a corundum crucible and heated under an air atmosphere (total flow rate 100 mL·min^−1^) from 30 to 800 °C at a heating rate of 10 °C·min^−1^.

Surface analyses by XPS were carried out in a system constructed by Prevac. The XPS spectra were collected using a monochromatized aluminum source Al Kα (E = 1486.6 eV) and a hemispherical analyzer (VG SCIENTA R3000, Newburyport, MA, USA). The binding energy scale for the conductive carbon samples was calibrated by referring to a position of Au 4f (E_b_ = 84.0 eV). The Shirley background and fitting with the mixed function of Gauss and Lorentz (GL = 30) were used during interpretation of the spectra in the CasaXPS software.

### 3.4. Adsorption Tests

The carbonized resol resin-derived materials were tested as adsorbents in the removal of phenol from aqueous solutions. In a typical test, 200 mg of a carbon material were added to 500 mL of an aqueous phenol solution with a concentration of 200 mg·L^−1^, kept in a thermostated flask (22.5 ± 0.2 °C), and placed on a magnetic stirrer (stirring rate = 200 rpm). The phenol concentration in samples withdrawn from the studied aqueous solution was spectrophotometrically determined using an Evolution 220 (Thermo Scientific, Waltham, MA, USA) dual-beam UV–Vis spectrometer equipped with a xenon lamp. The spectra were collected within a *λ* range of 200–400 nm, a resolution of 0.1 nm, and a scanning time of 60 min^−1^.

The efficiency of phenol adsorption was analyzed for the carbonized resol resins calculating adsorption capacity ($Q_{t}$) according to the following formula:

$$Q_{t}=\frac{V\cdot \left(C_{0}-C_{t}\right)}{m}$$

where V is the volume of a solution, m is an amount of an adsorbent (g), $C_{0}$ is the initial phenol concentration in the solution (mg·L^−1^), and $C_{t}$ is the phenol concentration in the solution after time *t* (mg·L^−1^).

Two models were applied to describe the phenol adsorption kinetics on the carbon materials: pseudo-first order (PFO) and PSO, expressed by the equations presented in Table 7.

Where $Q_{max}$ is the maximum amount of phenol adsorbed (mg/g), $k_{ad}^{\prime }$ is the pseudo-first order adsorption rate constant (s^−1^), $k_{ad}^{\prime \prime }$ is the pseudo-second order adsorption rate constant (g·mg^−1^·h^−1^), t is the time of the adsorption process (h), $t_{ref}$ is the longest operation time in adsorption process (h), $Q_{ref}$ is the adsorption capacity at time $t=\;t_{ref}$ (mg·g^−1^) and $R_{w}$ is the approaching equilibrium factor for PSO.

## 4. Conclusions

As shown in this study, the successful synthesis of spherical grains of resol resin is possible through the polycondensation of resorcinol and formaldehyde in a water–ethanol mixture using the adopted Stöber method. The proper choice of stabilizer plays a key role in the synthesis. The best results were achieved using poly(vinyl alcohol). The resin materials retained their spherical shape after carbonization at elevated temperatures. It was found that a hierarchical arrangement of pores in such grains provided perfect access to the micropores formed in the carbon structure. As a result, these materials showed excellent adsorption properties in the adsorption of phenol from aqueous solutions due to the preferential course of the process in micropores. Moreover, it was proved that adsorption, described by the pseudo-second order kinetics model, mainly occurred on graphite surfaces through the interaction of π electrons of a phenolic ring with the π electrons of a graphene layer. Hence, the progress of graphitization induced by increasing the carbonization temperature enhanced the adsorption capacity. On the other hand, the presence of heteroatoms hindered the adsorption of phenol molecules on the studied carbon materials due to blocking access to micropores and the limited participation of electron donor–acceptor complex- and hydrogen-bond-involving surface functionalities in the adsorption mechanism.

## Figures and Tables

**Table 1:** Textural parameters of the resol resin-derived carbon materials carbonized at 600 °C.

Carbon Sample	Mean Grain Size (nm)	S_BET_ (m^2^·g^−1^)	S_Langmuir_ (m^2^·g^−1^)	Total Pore Volume (cm^3^·g^−1^)	Mesopore Volume (cm^3^·g^−1^)	Micropore Volume (cm^3^·g^−1^)
52_non	750	640	725	0.345	0.043	0.226
52_PVP	200	511	604	0.259	0.051	0.176
52_X100	900	572	673	0.249	0.016	0.213
52_P123	120	613	742	0.403	0.160	0.206
52_PVA	460	598	706	0.301	0.042	0.220
61_PVA	230	577	702	0.507	0.066	0.209
131_PVA	100	583	685	0.502	0.130	0.192
52_HCl	1500	594	687	0.243	0.006	0.223

**Table 2:** Results of fitting the pseudo-first order and pseudo-second order kinetics models to experimental data of phenol adsorption on resol resin-derived carbon materials carbonized at 600 °C.

Carbon Sample	Pseudo-First Order Model			Pseudo-Second Order Model		
	Adsorption Capacity (mg·g^−1^)	$k_{ad}^{\prime }$ (h^−1^)	R^2^	Adsorption Capacity (mg·g^−1^)	$k_{ad}^{\prime \prime }$ (g·mg^−1^·h^−1^)	R^2^
52_non	96.42	2.361	0.9407	106.20	0.031	0.9667
52_PVP	56.08	4.024	0.9775	61.32	0.090	0.9899
52_X100	65.99	0.529	0.9214	76.47	0.008	0.9697
52_P123	74.39	9.724	0.9387	79.34	0.183	0.9831
52_PVA	86.22	6.413	0.9499	91.89	0.107	0.9805
61_PVA	85.22	6.052	0.9624	91.43	0.107	0.9617
131_PVA	69.01	20.60	0.8935	72.40	0.419	0.9930
52_HCl	81.20	1.567	0.9923	91.95	0.021	0.9981

**Table 3:** Contents of O and N species on the surface of the resol resin-derived carbon materials carbonized at different temperatures and subsequently modified in an oxidizing/reducing atmosphere.

Carbon Sample	O Species (at.%)				N Species (at.%)			
	C=O	O=C-O	C-O	N-O	Pyridinic	Pyrrolic	Graphitic	NO_x_
**Carbonization at 600 °C**								
52_PVA_600	1.1	0.7	2.6	-	0.8	0.4	0.2	-
52_PVA_600_ox	2.4	5.7	3.2	4.1	0.9	0.8	0.2	2.0
52_PVA_600_red	0.3	0.6	2.7	-	0.7	0.3	0.4	-
52_PVA_600_ox_red	1.1	0.9	3.1	-	2.0	2.1	-	-
**Carbonization at 800 °C**								
52_PVA_800	0.6	1.9	1.5	-	0.5	0.4	0.6	-
52_PVA_800_ox	2.3	2.8	0.6	2.3	0.4	0.3	0.5	1.1
52_PVA_800_red	0.2	0.3	0.3	-	0.5	0.3	0.7	-
52_PVA_800_ox_red	0.7	0.3	0.9	-	1.1	1.1	0.7	-
**Carbonization at 1050 °C**								
52_PVA_1050	0.3	0.3	-	-	-	0.2	0.4	-
52_PVA_1050_ox	2.3	1.8	0.6	1.2	0.2	0.2	0.3	0.6
52_PVA_1050_red	0.3	0.1	-	0.3	0.3	-	0.8	-
52_PVA_1050_ox_red	0.3	0.1	0.2	0.4	0.5	0.6	0.9	-

**Table 4:** Textural parameters of the resol resin-derived carbon materials carbonized at different temperatures and subsequently modified in an oxidizing/reducing atmosphere.

Carbon Sample	S_BET_ (m^2^·g^−1^)	S_Langmuir_ (m^2^·g^−1^)	Total Pore Volume (cm^3^·g^−1^)	Mesopore Volume (cm^3^·g^−1^)	Micropore Volume (cm^3^·g^−1^)
**Carbonization at 600 °C**					
52_PVA_600	598	706	0.301	0.042	0.220
52_PVA_600_ox	503	408	0.208	0.026	0.156
52_PVA_600_red	581	679	0.268	0.027	0.212
52_PVA_600_ox_red	619	706	0.286	0.030	0.224
**Carbonization at 800 °C**					
52_PVA_800	643	713	0.325	0.041	0.228
52_PVA_800_ox	475	551	0.225	0.023	0.156
52_PVA_800_red	552	642	0.260	0.030	0.203
52_PVA_800_ox_red	603	697	0.275	0.023	0.227
**Carbonization at 1050 °C**					
52_PVA_1050	490	620	0.284	0.036	0.192
52_PVA_1050_ox	545	636	0.256	0.026	0.201
52_PVA_1050_red	574	673	0.274	0.034	0.207
52_PVA_1050_ox_red	647	746	0.294	0.024	0.242

**Table 5:** Results of fitting the pseudo-second order kinetics model to the experimental data of phenol adsorption on the resol resin-derived carbon materials carbonized at different temperatures and subsequently modified in an oxidizing/reducing atmosphere.

Carbon Sample	Adsorption Capacity (mg·g^−1^)	$k_{ad}^{\prime \prime }\;$ (g·mg^−1^·h^−1^)	R^2^
**Carbonization at 600 °C**			
52_PVA_600	91.89	0.107	0.981
52_PVA_600_ox	52.86	0.002	0.829
52_PVA_600_red	91.59	0.047	0.991
52_PVA_600_ox_red	73.74	0.246	0.981
**Carbonization at 800 °C**			
52_PVA_800	113.33	0.180	0.985
52_PVA_800_ox	5.37	1.513	0.781
52_PVA_800_red	97.52	0.110	0.956
52_PVA_800_ox_red	95.40	0.108	0.949
**Carbonization at 1050 °C**			
52_PVA_1050	150.23	0.193	0.948
52_PVA_1050_ox	11.72	1.310	0.773
52_PVA_1050_red	57.91	0.179	0.954
52_PVA_1050_ox_red	114.76	0.102	0.990

**Table 6:** Composition of the reaction mixtures used in the syntheses of resol resins.

Sample Name	Stabilizer	Concentration (mM)	H_2_O:EtOH (*v:v*)	Initiator	Volume of Initiator Solution (mL)
52_non	none	-	5:2	NH_3_	2.5
52_X100	Triton X100	0.23	5:2	NH_3_	2.5
52_P123	Pluronic P123	0.33	5:2	NH_3_	2.5
52_PVP	PVP	0.05	5:2	NH_3_	2.5
52_PVA	PVA	0.01	5:2	NH_3_	2.5
61_PVA		0.01	6:1	NH_3_	2.5
131_PVA		0.01	13:1	NH_3_	2.5
52_HCl		0.01	5:2	HCl	3.6

**Table 7:** Model equations describing the kinetics of the phenol adsorption [50,51].

Kinetics Model	Non-Linear	Dimensionless
Pseudo-first order (PFO)	$Q_{t}=Q_{max}\cdot \left(1-\exp\left(-{k^{\prime }}_{ad}\cdot t\right)\right)$	QtQref=1−exp(−k·t/tref)1−exp(−k)
Pseudo-second order (PSO)	$Q_{t}=\frac{{k^{\prime \prime }}_{ad}\cdot Q_{max}^{2}\cdot t}{1+{k^{\prime \prime }}_{ad}\cdot Q_{max}\cdot t}$	QtQref=(t/tref)[1−(t/tref)]·Rw+t/tref

## Data Availability

The data presented in this study are available on request from the corresponding author.
